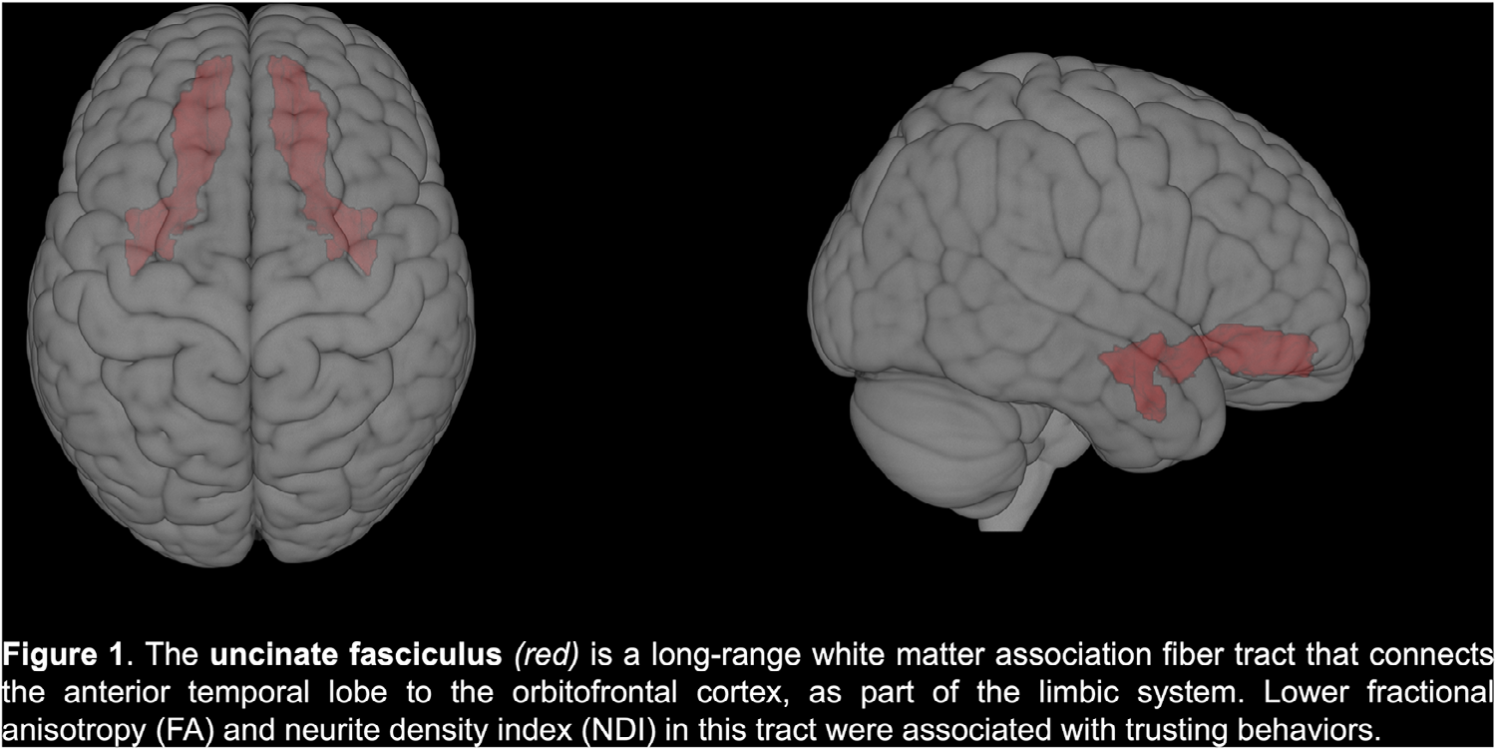# Trusting behavior associated with reduced white matter integrity in uncinate fasciculus in Alzheimer's disease and behavioral variant frontotemporal dementia

**DOI:** 10.1002/alz70857_105701

**Published:** 2025-12-25

**Authors:** Jayden J Lee, Derek B. Archer, Ryan Darby

**Affiliations:** ^1^ Department of Neurology, Vanderbilt University Medical Center, Nashville, TN, USA; ^2^ Vanderbilt Memory & Alzheimer's Center, Vanderbilt University Medical Center, Nashville, TN, USA

## Abstract

**Background:**

Interpersonal trust and cooperation are important aspects of prosocial behavior. Propensity to trust and willingness to cooperate with others may be altered in dementia, yet the neural underpinnings for these changes in trust remain poorly understood. The present study examines whether Alzheimer's disease (AD) and behavioral variant frontotemporal dementia (bvFTD) patients’ cooperative behavior during the Trust Game, a common experimental paradigm to quantitatively measure trust and trustworthiness in neuroeconomics, relates to white matter degeneration using diffusion tensor imaging (DTI) and neurite orientation dispersion and density imaging (NODDI) techniques.

**Method:**

Diffusion images (resolution: 2mm isotropic, *b*‐values: 0,1000s/mm^2^) were collected for 22 AD and 38 bvFTD patients. Preprocessing was completed using PreQual/Synb0‐DISCO pipelines. Participants played the Investor role in the Trust Game (TG), during which they were asked to invest money in two separate partners and receive a proportion of the profit in return. One partner returned a fair proportion of the investment (the *cooperative* partner) while the other partner did not (the *selfish* partner). White matter degeneration was assessed using fractional anisotropy (FA) and intra‐axonal volume fraction (ICVF) within well‐established white matter tracts. Linear regression models were performed to detect associations between diffusion metrics and TG measures while controlling for age and sex. Statistical significance was determined at *p* < 0.05, FWE‐corrected.

**Result:**

DTI/NODDI analysis revealed significant associations between participants' last investment in the TG with the cooperative partner and lower ICVF (*p* = 0.004) in the uncinate fasciculus (UF) white matter tract as well as between participants' change in investment over time with the cooperative partner and lower FA (*p* = 0.009) in the UF for the combined group. Additionally, bvFTD patients invested less money on average with the cooperative partner than AD patients (*p* = 0.01) and invested less over time with the cooperative partner.

**Conclusion:**

Our results suggest a distinct relationship between reduced white matter integrity in the UF and trusting behaviors in dementia patients and show that bvFTD patients are less cooperative overall than AD patients. These findings align with prior studies investigating the socioemotional sensitivity of the uncinate fasciculus, further suggesting that UF damage may contribute to behavioral symptoms such as impaired prosocial cooperation and trust.